# Emerging Strategies and Progress in the Medical Management of Marburg Virus Disease

**DOI:** 10.3390/pathogens14040322

**Published:** 2025-03-27

**Authors:** Sanctus Musafiri, Emmanuel Edwar Siddig, John Baptist Nkuranga, Athanase Rukundo, Tharcisse Mpunga, Augustin Sendegeya, Theogene Twagirumugabe, Ayman Ahmed, Claude Mambo Muvunyi

**Affiliations:** 1 University Teaching Hospital of Kigali (CHUK), Kigali KN 4 Ave, Rwanda; 2 School of Medicine and Pharmacy, College of Medicine and Health Sciences, University of Rwanda, Kigali 3900, Rwanda; 3 Rwanda Biomedical Centre, Kigali 7162, Rwanda; 4 Department of Pediatrics, King Faisal Hospital, Kigali 20093, Rwanda; 5 Department of Clinical Service, Ministry of Health, Kigali 84, Rwanda; 6 King Faisal Hospital, Kigali 20093, Rwanda; 7 Butare University Teaching Hospital, Huye P.O. Box 254, Rwanda; 8 Pan-Africa One Health Institute (PAOHI), Kigali 11KG ST203, Rwanda

**Keywords:** drug discovery, therapeutic development, clinical trials, vaccine development, preparedness, prevention, control of Marburg virus disease, case management, hemorrhagic fevers, filoviruses

## Abstract

During the current outbreak of Marburg virus disease (MVD) in Rwanda, we synthesized evidence from the literature to improve case management. Accordingly, experimental treatment was offered to patients under close follow-up. Remdesivir alone or in combination with monoclonal antibody treatment (MBP091) complemented with supportive care has improved the clinical outcomes of patients. Additionally, we have identified several experimental therapies currently under investigation, including antiviral drugs such as favipiravir, galidesivir, obeldesivir, and remdesivir, along with monoclonal and polyclonal antibodies (e.g., polyclonal IgG, monoclonal antibody MR-78-N; MR82-N; MR191-N; monoclonal antibodies MR186-YTE and MBP091). Furthermore, substantial progress is being made in vaccine development, with promising candidates including adenovirus-vectored vaccines, DNA vaccines, and the recombinant vesicular stomatitis virus (rVSV) vaccine. Moreover, innovative preventive and treatment strategies—such as synthetic hormones like estradiol benzoate, small interfering RNA (siRNA), interferon-β therapy, and phosphorodiamidate morpholino oligomers—are emerging as potential options for MVD management. Further investment is needed to accelerate research and optimize these therapeutics and preventive modalities. Additional epidemiological, preclinical, and clinical studies are warranted to generate the evidence required to inform policymaking, resource mobilization, and the implementation of cost-effective interventions for the prevention, control, and treatment of MVD.

## 1. Introduction

*Orthomarburgvirus marburgense*, commonly known as Marburg virus (MARV), is an emerging and highly pathogenic virus belonging to the *Orthomarburgvirus marburgense* species under the *Filoviridae* family, which is notorious for causing severe hemorrhagic fever with mortality rates that can exceed 80% in resource-variable settings [1,2,3,4]. Since its initial identification in 1967 following outbreaks among laboratory workers in Marburg and Frankfurt, Germany, and Belgrade, Yugoslavia (now Serbia), MARV has been linked to imported African green monkeys from Uganda, underscoring the zoonotic nature of the virus and its high transmissibility in specific environments [5]. From that period onward, MARV has caused sporadic outbreaks, primarily in sub-Saharan Africa, including Angola, Uganda, the Democratic Republic of Congo, United Republic of Tanzania, Ghana, Equatorial Guinea, and, currently, Rwanda [2]. The majority of these outbreaks have been characterized by high case fatality rates and significant public implications. As there are currently no licensed antiviral treatments specifically targeting MARV, the management of Marburg virus disease (MVD) mainly relies on supportive care and experimental therapeutics [6].

## 2. Route of Transmission and Clinical Presentation

**Figure 1:**
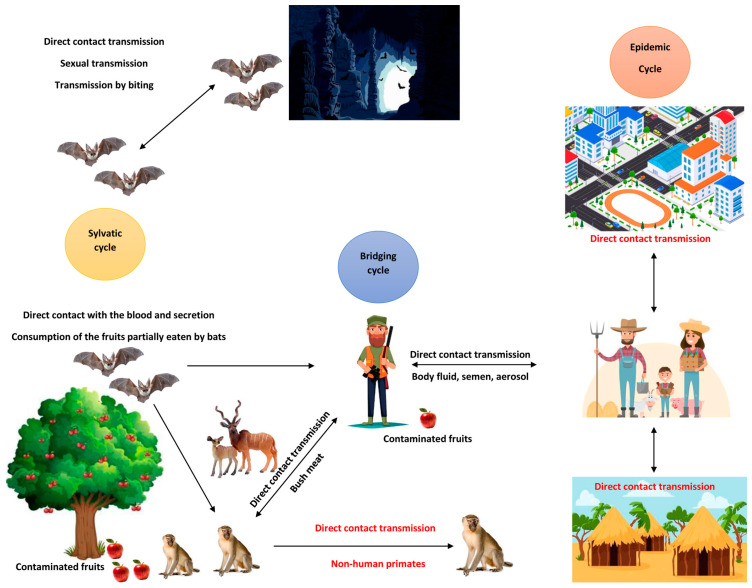
Illustration of the various transmission cycles of Marburg virus in different ecological settings (adopted from Muvunyi et al., 2024 [3]).

MARV transmission primarily occurs through mucosal surfaces, broken skin, or parenteral routes [3]. During outbreaks, the most common source of infection is direct contact with infected individuals or animals (Figure 1).

Parenteral exposure, particularly in healthcare environments, is one of the most lethal modes of transmission. Moreover, human-to-human transmission typically arises from direct exposure to various bodily fluids of infected individuals, including blood, saliva, sweat, feces, urine, tears, and semen—especially during the care of those who are infected. Moreover, traditional burial customs, which involve handling the bodies of deceased individuals, also pose a significant risk of transmission. Evidence from the 1967 outbreak also pointed to the possibility of sexual transmission during the recovery phase, as viral antigens were discovered in the semen of a patient with the infection. There is also documented evidence of mother-to-child transmission of MARV via breastfeeding. Additionally, in enclosed spaces with inadequate ventilation, the risk of MARV transmission is further increased through aerosol, droplets, and splashes of infected bodily fluids [3].

The clinical presentation of MVD includes fever, abdominal pain, vomiting, diarrhea, and, in severe cases, hemorrhagic manifestations occurring one to two weeks after initial symptoms, leading to severe dehydration and multi-organ failure [7]. Given the absence of specific antiviral therapies, the focus remains on meticulous supportive care, which is vital to improving patient outcomes and survival rates. Given the high mortality rates and severe public health implications associated with MVD, there is an urgent need for more effective therapeutic and preventive strategies. This review highlights recent advancements in the medical management of MVD, focusing on supportive care approaches, emerging experimental therapies, and ongoing vaccine research.

## 3. Marburg Virus Genomic Characteristics Related to Virus Entry, Transmission, and Targets for Treatment and Vaccine Strategies

**Figure 2:**
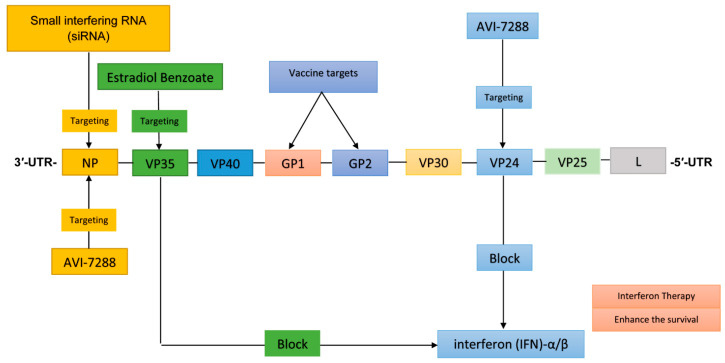
This illustration shows the interactions between different drugs and vaccines and genes in the Marburg virus genome, including therapeutic and vaccine targets. Different colors indicate pathways.

The genome of MARV is a single-stranded, negative-sense linear RNA that is not segmented and possesses inversely complementary 3′ and 5′ termini. It has a length of approximately 19 kb, and unlike some other viruses, MARV’s genome is not polyadenylated at the 3′ end and does not have a 5′ cap. The genome includes seven genes organized as follows: 3′-UTR-NP-VP35-VP40-GP1-GP2-VP30-VP24-VP25-L-5′-UTR (Figure 2). Each gene plays a specific role in the virus’s lifecycle: NP (nucleoprotein) is essential for encapsulating the RNA genome and forming the nucleocapsid, crucial for replication and transcription [8]. VP35 functions as a polymerase cofactor and inhibits interferon (IFN) signaling, aiding in nucleocapsid formation. VP40 facilitates viral budding and acts as an antagonist to the interferon response as well. GP1 and GP2 (glycoproteins) are involved in virus attachment to host cells, receptor binding, and membrane fusion during the entry process. VP30 contributes to the formation of the nucleocapsid. VP24 plays a role in the maturation of the nucleocapsid, and L (large protein) functions as the RNA-dependent RNA polymerase, responsible for viral genome replication and transcription [9].

Based on the different functions of these genes in the pathogenesis of MARV, several components present potential targets for antiviral strategies—VP35: targeting VP35 could lead to reduced viral replication through the disruption of its role as a polymerase cofactor and interferon antagonist. VP40: inhibiting VP40 may prevent viral budding and spread by interfering with its ability to facilitate these processes. Glycoprotein (GP1 and GP2) strategies that block the interaction of these glycoproteins with host receptors could prevent viral entry into host cells [10]. L protein inhibitors that target the RNA-dependent RNA polymerase function of the L protein and nucleoside analogs could be effective in halting viral replication and elongation. This review aims to encapsulate the current understanding of therapeutic options for MARV infection, highlighting emerging strategies and treatments that may pave the way for more effective medical management in the future.

## 4. Supportive Treatment

The primary approach to managing MVD is supportive care, which focuses on alleviating symptoms and preventing complications. This approach is crucial as there are currently no specific antiviral treatments approved for MVD [11]. Key components of supportive treatment include intravenous (IV) fluid therapies and electrolyte imbalance management, nutritional support, and the management of hemorrhagic manifestations with blood components and pro-coagulant products, with empirical broad-spectrum antibiotics administered to critically ill patients to prevent secondary bacterial infections due to immune dysregulations [7]. Patients with MVD frequently experience severe dehydration due to high fever, vomiting, and diarrhea. To address this, intravenous fluids and electrolyte replacement are essential for maintaining hemodynamic stability, supporting organ function, and preventing renal failure. However, the careful monitoring of fluid balance is crucial for guiding therapy and optimizing recovery. Ensuring adequate nutritional intake is critical, especially in patients suffering from significant weight loss and malnutrition [12]. A tailored nutritional plan, potentially supplemented with enteral feeding whenever possible, can aid in recovery and overall patient strength. Hemorrhagic symptoms can arise in some patients, necessitating the vigilant monitoring and proactive management of coagulopathy. In cases of severe bleeding, blood transfusions may be warranted to replenish lost components and preserve hemostasis [7,13].

## 5. Experimental Therapies

Currently, there are no robust clinically proven medical treatments for MVD, which has led to ongoing experimental therapies aimed at determining safe and effective treatment options [14]. These therapies are being rigorously tested in preclinical studies, primarily involving non-human primates, to evaluate their efficacy and potential cytotoxic effects before progressing to human clinical trials [15]. Experimental treatment strategies can be categorized into pre-exposure prophylaxis and post-exposure prophylaxis. Pre-exposure prophylaxis includes the development of vaccines aimed at providing immunity against MARV. Various candidates are under investigation, with the aim of establishing effective preventive measures for at-risk populations [15].

Post-exposure prophylaxis involves a range of experimental agents under investigation including antivirals, phosphorodiamidate morpholino oligomers (PMOs) [16], polyclonal and monoclonal antibodies [17], small interfering RNA (siRNA) [18], tumor necrosis factor (TNF) and interleukin-1 (IL-1) antagonists, and interferons [19]. These therapies are designed to inhibit virus replication, provide passive immunity, target viral RNA, reduce excessive inflammation, and enhance the immune response against MARV.

### 5.1. Antiviral Therapy

#### 5.1.1. Favipiravir (T-705)

T-705, also known as favipiravir, is a pyrazinecarboxamide derivative that functions as a nucleoside analog, inhibiting viral RNA polymerase or inducing lethal mutagenesis upon incorporation into viral RNA [20,21]. Notably, T-705 exhibits antiviral activity against a wide range of viruses in vitro and in vivo. In a mouse model of Ebola virus (EBOV) infection, T-705 demonstrated significant efficacy by reducing viremia, improving clinical and biochemical signs of the disease, and reducing the risk of complications [22]. The compound has also been proven effective against various influenza strains, including H1N1, H5N1, and the recently emerged H7N9 avian virus (Table 1). Additionally, T-705 has been shown to inhibit the replication of numerous other RNA viruses, such as arenaviruses (e.g., Junin, Machupo, and Pichinde viruses), flaviviruses (e.g., yellow fever and West Nile viruses), and enteroviruses (e.g., poliovirus, rhinovirus, and norovirus) [21]. Currently, T-705 is licensed for the treatment of influenza in Japan [23], and it was clinically investigated during the 2014 Ebola virus outbreak in Guinea [24].

In vivo (BALB/c mice) and in vitro (Vero E6 cells) model studies were conducted to investigate the efficacy of T-705 against MARV [25]. Their findings indicated that the oral administration of T-705, starting one to two days post-infection and continuing for eight days, resulted in complete survival in mice intraperitoneally infected with the mouse-adapted Angola strain of MARV. Even at lower doses or when administered later during infection (on days 3 or 4 post-infection), T-705 exhibited partial efficacy, resulting in the survival of at least half of the infected mice. This treatment correlated with observed reductions in infectious virus particles and viral RNA levels [25].

The effectiveness of favipiravir (T-705) in *cynomolgus macaques* infected with Ebola virus and the Angola strain of MARV was studied in 2018 (Table 1) [26]. The researchers found that treating the macaques with intravenous doses of favipiravir twice a day led to an 83% survival rate in those infected with MARV. In comparison, all untreated animals died. This study highlights the potential of T-705 as a therapy for serious viral infections like MARV [26].

#### 5.1.2. Galidesivir (BCX4430)

BCX4430, also known as galidesivir, is a promising nucleoside analog that specifically targets the RNA-dependent RNA polymerase (RdRp) of RNA viruses. It has shown significant antiviral activity against MARV, with effective concentrations (EC50) ranging from 4.4 μM to 6.7 μM. The concentrations required for 90% inhibition (EC90) were estimated between 10.5 μM and 16.1 μM across three distinct strains of MARV (Table 1). Importantly, the cytotoxic concentration (CC50) for all strains exceeds 200 μM, highlighting a favorable therapeutic index [27].

The in vivo efficacy of galidesivir against filoviruses was rigorously evaluated in a guinea pig model of MARV disease [28]. Significant protective effects were noted when treatment was administered via intraperitoneal injection at a dose of 15 mg/kg, given twice daily, and initiated within 48 h of viral challenge. Additionally, galidesivir remained effective even when treatment commenced within 72 h following exposure to aerosolized viral particles, indicating its potential as a viable post-exposure therapeutic agent [27].

Following this initial success in guinea pigs, the group also used a *cynomolgus macaque* model of MARV disease. This non-human primate (NHP) model accurately reflects the pathology associated with fatal human cases of MARV infection. In this setting, galidesivir demonstrated robust antiviral activity when administered at a higher dose of 50 mg/kg, twice daily, starting either 24 or 48 h after viral challenge (Table 1). This treatment regimen led to a significant reduction in viremia and other disease-related symptoms and complications, resulting in a remarkable 100% survival rate among the treated animals [27]. These findings collectively underscore the significant therapeutic potential of galidesivir as an antiviral agent against MARV, particularly in post-exposure scenarios.

#### 5.1.3. Remdesivir (GS-5734)

Remdesivir (GS-5734) is a monophosphoramidate prodrug of an adenosine nucleoside analog that has demonstrated therapeutic efficacy in non-human primates (NHPs) against various lineages of RNA viruses, including those from the *Filoviridae*, *Paramyxoviridae*, and *Coronaviridae* families (Table 1). Notably, it has shown in vitro inhibitory activity against Sudan virus (SUDV). Remdesivir functions by restricting viral replication through mechanisms that hinder the synthesis of viral RNA (vRNA). This occurs via RNA-dependent RNA polymerase through delayed chain termination and template-mediated inhibition [29,30].

In a study aimed at evaluating remdesivir’s potential role in treating MVD, researchers established a *cynomolgus macaque* model, dividing the subjects into four groups of three male and three female monkeys each [30]. All groups were inoculated with MARV. The control group received no treatment, while two treatment groups were administered a loading dose of 10 mg/kg on days 4 and 5, followed by a daily maintenance dose of 5 mg/kg for 11 days. The fourth group received a daily dose of 5 mg/kg from days 5 to 16. The control animals developed acute MVD symptoms, and all either succumbed or were euthanized between days 7 and 9 post-inoculation. In contrast, remdesivir treatment resulted in a statistically significant increase in survival rate to 83% in both treatment groups receiving the 10 or 5 mg/kg regimen, regardless of whether treatment commenced on day 4 or 5 post-exposure, and a 50% survival rate in the group treated with 5 mg/kg starting from day 5. Additionally, remdesivir-treated animals showed improved clinical outcomes, including lower plasma viral RNA levels, enhanced kidney and liver function markers, and reduced signs of coagulopathy, compared to those receiving the vehicle control, underscoring its therapeutic potential for MVD in humans [30].

Further investigations assessed the efficacy of remdesivir against MARV using a rhesus macaque model [31]. In this study, five monkeys infected with the Angola variant of MARV received an initial loading dose of 10 mg/kg intravenously, followed by a maintenance dose of 5 mg/kg for 12 consecutive days. All monkeys exhibited symptoms indicative of MVD, such as fever (lasting 5 to 8 days), decreased appetite, rashes, and diarrhea. Remarkably, four out of the five animals were completely cured and tested free of MARV by day 19 post-infection.

Of note, remdesivir is recognized as the only antiviral therapy authorized for use in treating patients with COVID-19, demonstrating significant clinical benefits in several phase 3 clinical trials (Table 1). This supports its potential as a promising treatment option for MVD, warranting further clinical evaluation and investigation. To date, remdesivir is only available as injectable product, and it could be interesting to have an oral alternative medication for post-exposure prophylactic and therapeutic purposes since outbreaks often occur in resource-limited settings, where injectable therapy can be a substantial logistical challenge.

#### 5.1.4. Obeldesivir (GS-5245)

Obeldesivir (GS-5245) is an investigational nucleoside prodrug that is metabolized in cells and tissues to the same active GS-441524 triphosphate metabolite as remdesivir and thus exerts an identical antiviral mechanism of action [32,33,34]. Obeldesivir (ODV) is an oral antiviral drug of the parent nucleoside of remdesivir and has been studied for COVID-19 in phase 3 clinical trials [33]. Experimental studies have also revealed its effectiveness against the Ebola, Sudan, and Marburg viruses [33,35]. Experiments on NHPs infected with Sudan virus have demonstrated that ODV treatment started on day 1 post-exposure as a single daily dose for 10 days provided complete protection against SARS-CoV-2 lethal infection. Additionally, it has shown a delayed onset of inflammation and features of activation of the immune system [36].

Obeldesivir’s potential has opened opportunities for its experimentation in the post-exposure prophylaxis and treatment of filovirus infection. As an oral drug with an immediate effect in comparison with vaccines, it offers better opportunities for the management of filovirus outbreaks that happen mainly in remote areas of resource-limited settings in Africa with important challenges in the conservation and supply chains of medicines and vaccines. It could be interesting to explore whether the administration of obeldesivir can quicken the clearance of viruses from reproductive organs, especially when tested among survivors of MVD and EVD, limiting the risk of sexually transmitted infections that can trigger subsequent outbreaks [37].

### 5.2. Monoclonal and Polyclonal Antibodies

The use of antibodies for the post-exposure treatment of viral hemorrhagic fever infections has yielded varied results [38,39]. Passive antibody transfer from survivors of Junin virus or Lassa virus infections has shown effectiveness, especially when therapy is administered promptly after exposure [38,39]. However, similar approaches for filovirus infections, such as those caused by Ebola virus (EBOV), have not been as successful [40].

#### 5.2.1. Polyclonal Antibodies

During a significant outbreak of EBOV in Kikwit, Democratic Republic of the Congo, seven out of eight symptomatic patients who tested positive for EBOV antigens in their blood survived after receiving whole blood transfusions from convalescent survivors [41]. The survival rate of 87.5% in this group was notably lower than the overall outbreak case fatality rate of 80% (Table 2). However, it is challenging to assess the specific contribution of antibodies to this observed survival, as the patients received whole blood—encompassing both antibodies and other components—along with supportive medical care [7,42].

Studies have indicated that polyclonal IgG antibodies can confer protection against MARV [43]. Interestingly, the administration of species-matched polyclonal IgG provided complete protection in NHPs challenged with filoviruses, including Ebola and MARV (Table 2). Remarkably, this protective effect was observed even when treatment was introduced as late as 48 h post-exposure. In the study, NHPs infected with MARV received treatments of virus-specific IgG just 15 to 30 min after exposure, with additional doses on days 4 and 8. This early intervention resulted in complete protection, and the treated subjects did not show any sign of disease or detectable viremia (Table 2).

#### 5.2.2. Monoclonal Antibodies

Furthermore, MARV-specific IgM antibody responses were elicited, indicating that the NHPs developed an immune response to viral replication, as all macaques survived subsequent re-challenges with MARV. Subsequent experiments involved infecting NHPs with either MARV or EBOV, with treatments initiated 48 h following exposure, alongside further doses on days 4 and 8. The delayed administration of polyclonal IgG still conferred protection against both types of infections. In these trials, two out of three IgG-treated NHPs exhibited no clinical symptoms, while the third experienced mild, delayed symptoms followed by complete recovery within days [43]. Since this study was published, several studies have been conducted to evaluate the therapeutic efficacy of various monoclonal antibodies against MARV. One of these studies demonstrated that MR191-N can result in a survival rate of up to 100% for rhesus macaques infected with Marburg or Ravn virus if treatment begins within five days of inoculation [44]. MBP091 is a newly developed monoclonal antibody derived from a survivor of MVD, specifically from the MR191-N clone. This antibody belongs to the IgG1 lambda subclass and has been developed and tested by Mapp Biopharmaceutical Company. MBP091 is designed to bind to the GP of MARV, neutralizing the virus by occupying a conserved receptor-binding site. Its effectiveness has been demonstrated in various animal models, including guinea pigs and rhesus macaques, where it has shown promising results against MARV infections. Currently, MBP091 is undergoing clinical trials in Rwanda, reflecting its potential as a therapeutic option for combating MVD [45,46]. A recent study investigated the therapeutic efficacy of a half-life-extended version of the MARV monoclonal antibody MR186, known as MR186-YTE. This afucosylated variant enhances antibody-dependent cellular cytotoxicity and promotes neutrophil activation and phagocytosis. In a rhesus macaque model of the MARV Angola variant, a single 100 mg/kg dose of MR186-YTE was administered 5 days post-inoculation. The results demonstrated successful recovery from lethal Marburg virus disease (MVD) in all four treated animals. However, when MR186-YTE was administered at 6 days post-inoculation, it uniformly failed to protect the animals from lethal infection [29]. Interestingly, the combination of MR186-YTE with remdesivir, when initiated at 6 days post-infection (dpi), demonstrated significant protection, achieving an impressive 80% efficacy. These findings highlight the potential benefits of exploring combination therapy for patients with advanced filovirus disease [29]. These findings extend the small but growing body of evidence that mAbs can impart a therapeutic benefit during advanced stages of disease with highly virulent viruses and could be useful in epidemic settings.

### 5.3. Marburg Virus Vaccine

Currently, there is no vaccine for MARV that has received regulatory approval for public use. Importantly, vaccines developed for Ebola do not provide cross-protection against MARV, though several vaccine candidates have shown promise in studies involving *cynomolgus macaques*, demonstrating protective efficacy against both Marburg and Ravn viruses. Among the active candidates, three vaccines—cAd3, MVA-BN-Filo, and MARV DNA—are in phase 1 clinical trials, with the MVA-BN-Filo vaccine also scheduled to progress into a phase 2/3 trial [47]. Various platforms, including recombinant vesicular stomatitis virus (rVSV), virus-like particles (VLPs), adenoviral vectors, and DNA vaccines, have shown protective effects in non-human primates (NHPs) [48,49,50,51].

#### 5.3.1. Adenovirus-Vectored Vaccines

Adenovirus-vectored vaccines have been explored primarily for Ebola, with limited focus on MARV. The recombinant adenovirus serotype 5 (rAd5) has emerged as the leading vector for glycoprotein (GP) vaccines (Table 3). In one study involving macaques administered a single dose of an rAd5 vaccine expressing the MARV Angola GP, all subjects remained healthy after being challenged with the homologous virus four weeks later. Similar protection was observed in another experiment where four macaques received three doses of MARV Angola GP DNA in a prime–boost regimen prior to vaccination [52].

The CAdVax platform, a sophisticated adenovirus-based vaccine, incorporates five antigens, including glycoproteins and nucleoproteins from EBOV, SUDV, and MARV, specifically from the Ravn, Musoke, and Ci67 strains. In clinical trials, *Cynomolgus macaques* were administered this vaccine using a prime–boost strategy and were subsequently challenged with EBOV, SUDV, and MARV (Musoke and Ci67 strains). Remarkably, all monkeys developed antibodies against all the tested filoviruses, and none showed any clinical symptoms of illness [53].

Nonetheless, the use of adenoviral vectors in vaccination programs has been hampered by pre-existing immunity within human populations [54]. To circumvent this limitation, researchers have begun utilizing less-common adenoviral serotypes and exploring alternative administration routes, such as oral or nasal delivery. Vaccines vectored by Ad26 and Ad35 have demonstrated lower levels of protection than rAd5 [55]. However, an Ad26-vectored vaccine was able to protect three out of four NHPs following an Ebola challenge when paired with a prime–boost strategy alongside an Ad35-vectored vaccine. Additionally, chimpanzee adenovirus 3 (Chad3) has been evaluated as a GP vector, with four animals surviving post-vaccination challenges against Ebola virus, though long-lasting immunity was not established [56].

A combined cAd3 prime vaccination followed by a boost using Modified Vaccinia Ankara (MVA) eight weeks later led to complete protection against Ebola in trials conducted on NHPs [57]. While similar studies with Marburg are still pending, clinical trials for analogous vaccines are ongoing [58].

A phase 1 clinical trial was conducted to investigate the cAd3 Marburg vaccine, in an open-label, dose-escalation study at the Walter Reed Army Institute of Research Clinical Trials Center in the USA. The trial enrolled 40 healthy adults aged 18 to 50 years, who were assigned to receive a single intramuscular dose of the cAd3 Marburg vaccine. Primary safety endpoints included assessing reactogenicity for the first 7 days and monitoring all adverse events for 28 days post-vaccination. Secondary immunogenicity endpoints focused on evaluating binding antibody responses and T-cell responses to the MARV glycoprotein, as well as assessing neutralizing antibody responses against the cAd3 vector 4 weeks after vaccination. The vaccine demonstrated a favorable safety profile, being well tolerated and immunogenic. Notably, there were no serious adverse events related to the vaccination [59].

Mild-to-moderate reactogenicity was reported following vaccination, with the most common symptoms being injection site pain and tenderness (27 participants, or 68%), malaise (18 participants, or 45%), headache (17 participants, or 43%), and myalgia (14 participants, or 35%). Glycoprotein-specific antibodies were induced in 38 (95%) of the 40 participants 4 weeks post-vaccination, and these antibody levels remained significantly elevated at 48 weeks. Additionally, T-cell responses to the glycoprotein insertion and neutralizing responses against the cAd3 vector were also significantly increased 4 weeks post-vaccination [59].

#### 5.3.2. DNA Vaccines

DNA vaccines targeting filoviruses demonstrate promising safety profiles in NHP trials and are easy to produce, effectively eliciting both humoral and cellular immune responses [60,61]. However, their immunogenicity in clinical settings has been somewhat limited. Studies utilizing DNA vaccines that include the glycoprotein (GP) from both MARV Musoke and MARV Angola have effectively elicited antibody responses in *Cynomolgus macaques*. This response afforded a degree of protection against challenges with the same strain of the virus. However, animals that were vaccinated still exhibited clinical symptoms of the disease, indicating that the presence of IgG antibodies alone may not be enough to fully control the infection [62].

Combining DNA vaccines with a prime–boost strategy, such as pairing them with adenoviral vectors, has demonstrated increased effectiveness. One notable example is the plasmid vaccine VRC MARDNA025-00-VP, which expresses MARV Angola DNA and has successfully completed phase 1 testing. This trial involved 10 participants and was designed to evaluate the safety and immunogenicity of the DNA vaccine. Participants received 4 mg administered intramuscularly via a Biojector at weeks 0, 4, and 8, followed by a homologous boost at or after week 32. Safety evaluations included monitoring solicited reactogenicity and assessing coagulation parameters. The primary immune response was evaluated using a glycoprotein-specific enzyme-linked immunosorbent assay (ELISA) [49]. The results indicated that the vaccine was well tolerated, with recipients experiencing only mild pain and redness at the injection site, as well as mild malaise, myalgia, and headache. Notably, 90% of participants developed antibody responses to the vaccine, highlighting its immunogenic potential [49].

#### 5.3.3. Recombinant Vesicular Stomatitis Virus (rVSV) Vaccine

The recombinant vesicular stomatitis virus (rVSV) platform has emerged as a promising candidate for the development of vaccines against MARV [63]. This advanced approach utilizes a replication-competent version of VSV that has been genetically modified to express the glycoprotein (GP) of MARV in place of its native glycoprotein.

In recent preclinical trials, *cynomolgus* monkeys were administered a single intramuscular dose of the rVSVΔG MARV Musoke GP vaccine, demonstrating complete protection against a subsequent high-dose (1000 PFU) intramuscular challenge with homologous MARV administered 28 days post-vaccination. Importantly, the vaccinated monkeys exhibited sustained immunity, indicated by resisting challenge with the 1967 strain of MARV 113 days later [48].

Furthermore, the rVSVΔG MARV Musoke GP vaccine showed effectiveness against the genetically distinct Ravn strain and the highly virulent Angola strain of MARV. These findings indicate the vaccine’s potential for cross-protection among diverse MARV strains [64]. Subsequent studies reinforced these promising results, revealing that a single vaccination completely shielded *cynomolgus* monkeys from a homologous aerosol challenge of MARV conducted 28 days after vaccination.

Additionally, a trivalent vaccine utilizing three VSV vectors has been developed, incorporating antigens for MARV, EBOV, and Sudan virus (SUDV). When administered to NHPs, this vaccine elicited robust antibody responses to all three components, achieving 100% cross-protection against MARV, EBOV, and SUDV 28 days post-vaccination, with only one animal displaying viremia [65].

Interestingly, in addition to its utility as a preventive vaccine, the rVSVΔG vaccine platform has also been used as a post-exposure treatment for filovirus infections. The treatment of rhesus monkeys with rVSVΔG MARV Musoke GP shortly after a homologous high-dose MARV challenge resulted in the complete protection of all animals from clinical illness and death [66].

Moreover, an open-label, cluster-randomized ring vaccination trial (Ebola ça Suffit!) conducted during the 2015 Ebola virus outbreak in communities in Conakry, Guinea, and surrounding prefectures, as well as in Tomkolili and Bombali in Sierra Leone, demonstrated remarkable efficacy. The estimated vaccine efficacy was found to be 100%, with all vaccine recipients followed for 84 days. Out of 5837 individuals, 3149 (53.9%) reported at least one adverse event within 14 days of vaccination; however, these events were predominantly mild, with headache (25.4%), fatigue (18.9%), and muscle pain (13.1%) being the most common. The rVSV-ZEBOV vaccine offers substantial protection against Ebola virus disease, as evidenced by the absence of cases among vaccinated individuals from day 10 post-vaccination in both randomized and non-randomized clusters [67]. Overall, these findings highlight the significant potential of the rVSV platform in developing effective vaccines against MVD and underscore its utility in regions where MARV, EBOV, and SUDV are endemic, providing a comprehensive immunization strategy against these circulating viruses.

## 6. New Target for New Hope

### 6.1. Estradiol Benzoate (EB)

Estradiol benzoate (EB) is a synthetic hormone that acts as a pro-estradiol, selectively binding to estrogen receptors (ERs), including the subtypes ERα and ERβ [68]. It is widely used in hormone therapy, the treatment of gynecological disorders, and the management of prostate cancer, and it was discovered that it demonstrates promising antiviral properties. Recent research has highlighted EB’s ability to inhibit the replication of several viruses, including SARS-CoV-2 and hepatitis B virus, primarily by blocking their entry into host cells. Specifically, EB disrupts the fusion process of SARS-CoV-2 by targeting and interfering with a critical viral structure [68,69,70].

In a chemoinformatic investigation using a virtual screening approach, researchers evaluated 2042 natural compounds for potential interactions with the VP35 protein of MARV [71]. The results indicated that EB stands out as a potential therapeutic option. Studies have shown that EB interacts significantly with the viral protein VP35 through covalent bonds, demonstrating a strong binding affinity. Molecular simulations further revealed a stable interaction between EB and VP35, along with favorable binding energy, suggesting that EB may effectively inhibit the function of VP35, i.e., viral replications and immune evasion [71]. Moreover, assessments of EB’s safety and drug-like properties indicate that it meets essential criteria for therapeutic use, including suitable molecular weight and acceptable toxicological profiles. These findings underscore estradiol benzoate as a promising candidate for the treatment of MARV infections, given its effective binding to and inhibition of key viral proteins [71]. However, preclinical studies are necessary to further investigate its therapeutic potential and efficacy.

### 6.2. Small Interfering RNA (siRNA)

RNA interference (RNAi) is a naturally occurring biological mechanism that plays a pivotal role in the regulation of gene expression. Small interfering RNA (siRNA) has proven effective in inhibiting the replication of various hemorrhagic fever viruses in vitro, including yellow fever virus, dengue, MARV, Lassa virus, Junin virus, and Ebola virus [72,73,74]. Utilizing siRNA as a post-exposure therapeutic strategy offers several advantages, such as a rapid design capability for emerging viral threats, established large-scale manufacturing processes, and a clear mechanism of action that can be empirically validated both in vitro and in vivo [75]. However, one of the main obstacles in developing siRNA therapeutics lies in ensuring the safe and effective delivery of these molecules. This requires the use of drug delivery vehicles that protect siRNA from degradation by nucleases and facilitate its uptake by target cells and tissues [75]. An initial study successfully identified a specific siRNA, NP-718 m, targeting the nucleoprotein of MARV. When encapsulated in lipid nanoparticles, which enhance cellular entry by fusing preferentially with the endosomal membrane, NP-718 m effectively inhibited MARV replication in vitro and demonstrated broad protective effects against three strains (Angola, Ci67, and Ravn) of the virus in guinea pig models [76].

Following these encouraging results, further investigation of NP-718 encapsulated in lipid nanoparticles (NP-718-LNP) was conducted in a study involving Marburg-infected rhesus macaques [77]. In this study, twenty-one macaques were challenged with the MARV Angola strain at doses ranging from 1000 to 1775 plaque-forming units (pfus) and subsequently treated with seven daily intravenous doses of NP-718-LNP. Administration commenced 30 to 45 min after infection, followed by treatments at 24, 48, and 72 h post-infection. Remarkably, all sixteen macaques that received the treatment survived, exhibiting significantly less severe clinical symptoms and markedly lower levels of viremia compared to untreated controls. These findings underscore the therapeutic potential of siRNA-based interventions as a viable strategy for combating MVD.

### 6.3. Interferon-β Therapy

MARV and EBOV inhibit interferon (IFN)-alpha/beta signaling through different mechanisms [78]. EBOV uses its VP24 protein to block the nuclear accumulation of tyrosine-phosphorylated STAT1. In contrast, MARV obstructs the tyrosine phosphorylation of both STAT1 and STAT2 induced by IFN-alpha/beta [79]. Recent studies have revealed that MARV also interferes with IFN-gamma-induced STAT phosphorylation and inhibits the activation of upstream Janus (Jak) family kinases involved in these signaling pathways. Notably, the MARV matrix protein VP40 has been found to antagonize Jak and STAT tyrosine phosphorylation, thereby limiting gene expression in response to both IFN-alpha/beta and IFN-gamma and disrupting the establishment of an antiviral state associated with IFN-alpha/beta [78].

In a study investigating the protective effects of IFN-β against MARV infection, four macaques were inoculated with a lethal dose of MARV Musoke. Three macaques received daily IFN-β treatments at a dose of 35 µg/kg starting one hour after infection for a duration of 14 days, while one macaque received saline as a control. Remarkably, one of the treated macaques displayed complete immunity, surviving beyond the study endpoint of 28 days and ultimately being euthanized 112 days post-infection, with no evidence of MARV persistence observed during autopsy. When the survivor was excluded from the analysis, the mean survival time for the IFN-β group was 14.5 days, compared to 10.8 days for the control group, which included five historical controls. However, when the survivor was taken into consideration, the difference in mean survival times between the treated and control groups was statistically significant (*p* = 0.0186, Wilcoxon rank-sum test).

The administration of IFN-β was associated with significant trends, including reduced viremia, decreased peripheral leukocyte counts, and an increased percentage of peripheral lymphocytes. The long-term survivor exhibited significant suppression of systemic cytokine production, and the intensive IFN-β treatment did not correlate with elevated serum markers suggestive of liver or kidney damage [19]. This study demonstrated that IFN-β can be used as an adjuvant therapeutic option for MARV infection.

### 6.4. Phosphorodiamidate Morpholino Oligomers

AVI-7288 is a 23-mer antisense phosphorodiamidate morpholino oligomer (PMO) designed for the post-exposure prophylaxis of MARV infection, specifically targeting the messenger RNA sequence of the MARV nucleoprotein to block its translation [80]. Studies in non-human primates, which closely mimic the human disease course, showed that treatment with AVI-7288 resulted in significant survival rates, particularly when administered at a dose of 15 mg per kilogram per day for 14 days up to 4 days post-infection, achieving survival rates of 83% to 100% compared to 0% in control subjects [81]. Initial estimates suggest a protective dose for humans of 9.6 mg per kilogram per day, supported by Monte Carlo simulations indicating 11 mg per kilogram per day for optimal efficacy. Notably, in multiple-ascending-dose studies, no safety concerns were observed at doses up to 16 mg per kilogram per day, with no significant toxic effects noted even at levels exceeding the estimated protective dose [81]. Overall, AVI-7288 demonstrates promising efficacy and safety as a prophylactic treatment following potential exposure to MARV.

### 6.5. Eritoran Tetrasodium (E5564)

Eritoran is a Toll-like receptor 4 (TLR4) antagonist that presents a promising therapeutic approach to addressing the severe complications associated with MVD and other forms of septic shock, particularly those induced by Gram-negative bacteria, which is currently being tested in a phase 3 clinical trial enrolling patients with severe sepsis (ClinicalTrials.gov identifier: NCT00334828) [82,83,84]. MVD exhibits many characteristics akin to septic shock, including acute symptoms such as fever, headache, and muscle pain. These symptoms can progress to more severe manifestations, including significant bleeding and coagulopathies, such as disseminated intravascular coagulation (DIC) [7]. There is also notable immune dysfunction characterized by lymphopenia and systemic inflammation, attributed to a massive, uncontrolled release of inflammatory mediators, commonly referred to as a “cytokine storm” [85,86,87]. In both MVD and bacterial sepsis, these inflammatory processes can lead to endothelial dysfunction and organ failure, complicating patient outcomes [88,89].

The pathophysiological response to Gram-negative bacterial infection is primarily driven by the production of lipopolysaccharides (LPSs), which activate the TLR4 pathway [90,91]. Upon exposure to LPSs, host cells produce reactive oxygen species as part of the normal inflammatory response, exacerbating inflammatory signaling via TLR4 activation [92,93]. Studies show that TLR4^−/−^ mice exhibit resistance to lethal influenza virus infections, underscoring the importance of this pathway in inflammatory responses [94]. Eritoran functions by mimicking lipid A, a component of LPSs, binding to the myeloid differentiation factor 2 (MD2) associated with TLR4 without inducing the receptor’s dimerization and subsequent activation [95,96]. This blockade of TLR4 signaling may effectively dampen the inflammatory response associated with viral infections and sepsis [97].

In preclinical research, eritoran has shown potential in mouse models infected with EBOV [84]. The daily administration of eritoran resulted in a reduction in clinical disease signs and unexpectedly decreased viral titers. Although the treated mice exhibited lymphopenia, characterized by reduced CD3+ T lymphocytes, there was a significant reduction in granulocytosis and the levels of various cytokines and chemokines. This suggests that eritoran treatment could mitigate the severity of the cytokine storm typically observed during severe viral infections [84]. In the same study, the researchers also targeted MARV, with mice treated with eritoran for ten days after infection exhibiting a survival rate of 90%, compared to only 20% in the placebo group [84]. This highlights eritoran’s potential role in reducing inflammation and improving survival rates in lethal viral infections. The findings regarding eritoran’s effectiveness in preclinical models indicate that TLR4 antagonism could serve as a valuable therapeutic strategy for managing the inflammatory responses associated with MVD and similar viral infections. However, further investigations are warranted to explore the safety, efficacy, and mechanistic nuances of eritoran in non-human primate models before translating these findings to clinical settings.

### 6.6. Glycoprotein (GP) Receptor Antagonists

Glycoprotein (GP) receptors present a compelling target for developing drug therapies against different viruses due to their crucial role in the viral entry process, a vital step for infection [98,99,100]. Recent research has explored chemical libraries of FDA-approved drugs, identifying several GP receptor antagonists that effectively block the entry of both EBOV and MARV by targeting different types of GP receptors, including histamine, serotonin, muscarinic acetylcholine, and adrenergic receptors [98]. These antagonists demonstrate significant antiviral activity using cell lines, inhibiting viral replication without causing considerable harm to host cells. Notably, the mechanism of these antagonists appears to interfere with the virus later in the entry process, after initial attachment but before the fusion of viral and host cell membranes [98]. This suggests that GP receptors may play a direct or indirect role in the entry of these viruses. With about half of clinically approved drugs targeting GP receptors, there is a wealth of existing compounds that can be repurposed for MVD treatment. The established safety profiles and diverse structures of these GP receptor antagonists highlight their potential as broad-spectrum antiviral therapies, offering a novel and promising approach to combatting MVD and enhancing current treatment strategies for this severe disease.

## 7. Case Management of MVD During the Recent Outbreak in Rwanda

Since September 27, 2024, an outbreak of MVD has erupted in Rwanda. The outbreak has primarily affected healthcare providers; however, through rapid response, it has resulted in 66 cases. The demographic breakdown reveals that the majority of cases involve males (68.18%) compared to females (31.82%), with the most impacted age group being 30–39 years (30 cases), followed by 20–29 years (20 cases), 40–49 years (11 cases), and 50–59 years (3 cases), and the least affected group being individuals under 20 years (2 cases) [101]. Despite the novel emergence of MVD in Rwanda, the prepared health system has been resilient and absorbed the shock of the sudden development of the disease outbreak. This has mainly been achieved through the investment made in early preparedness by building diagnostic capacity, surveillance, and contact tracking, and effective infection prevention and control (IPC) as well as case management [102]. In addition to strengthening IPC protocols, particularly in healthcare facilities, and other preventive measures including vaccinating healthcare providers and community health workers using the experimental Sabin vaccine, experimental treatment protocols were implemented under the emergency response framework [2,103,104]. Accordingly, remdesivir was administered alone and in combination with monoclonal antibody treatment (MBP091) for confirmed patients [2,104]. Remdesivir was also provided as a post-exposure prophylaxis (PEP) measure for seemingly healthy contacts of confirmed cases including those that tested negative for MARV [2,104]. Furthermore, this experimental treatment was complemented with the intensive care of patients under treatment.

This has resulted in an over 75% recovery rate with only 15 deaths related to MVD reported during this outbreak, representing a case fatality rate of 22.7%, which is very low in comparison with that of other outbreaks of MVD [2]. These effective prevention and case management measures have prevented the establishment of transmission among the community.

## 8. Future Directions and Conclusions

While the approved medical treatment options for MARV remain limited, promising experimental therapies and supportive care strategies show potential for improving outcomes.

The sudden eruption of an MVD outbreak in Rwanda is an urgent call for the global health community to invest in research into and the development of safe and cost-effective vaccine and therapeutics for MVD. Considering the initial success of treatment with remdesivir or remdesivir + monoclonal antibody treatment (MBP091), further investigations might help in progressing their official approval for MVD.

Considering that MVD outbreaks are increasingly occurring, much more investment is needed for disease-related research and development to improve the availability of safe and cost-effective prophylactic and therapeutic interventions. Currently, options for prophylaxis including polyclonal antibodies have been developed and administered to help prevent the onset of the disease. Several vaccine candidates are undergoing investigation, including CADVax, cAd3, and various DNA vaccines, such as the plasmid vaccine VRC-MMRNDA625-00-VP. In terms of treatment, a number of antiviral agents have shown potential efficacy against Marburg virus. These include favipiravir and remdesivir, which are both known for their broad-spectrum antiviral properties. Additionally, obeldesivir is being explored as a therapeutic option. Ongoing research aims to refine these treatments and further investigate their efficacy and safety profiles. Improving the understanding of the Marburg virus and its clinical management is critical for improving patient outcomes and responses to outbreaks.

Ongoing research is imperative to develop effective antiviral treatments and vaccines to combat MARV infections. Treatment with remdesivir alone and in combination with monoclonal antibody treatment (MBP091) has been implemented in Rwanda, and the result seems promising, yet effectiveness can only be confirmed with a larger sample size supported by immunological and systematic analyses. Nonetheless, these treatment modalities could be implemented experimentally during emergencies. It is worth noting that MVD survivors still carry the risk of transmission to sexual partners, and an experimental treatment such as remdesivir, or its oral analog obeldesivir, deserves a trial. The global health community must remain vigilant and prepared for future outbreaks, ensuring that research and preparedness efforts continue to evolve. More investment and collaboration are needed from the stakeholders of Global Health Security to support research and development to improve the generation of evidence and development of diagnostic tools, safe cost-effective treatment modalities, and cost-effective preventive and control measures including vaccines.

## Figures and Tables

**Table 1:** Summary of antiviral therapies for MARV, categorized by their type, mode of action, efficacy, other potential pathogen targets, and current regulatory phase toward their approval.

Antiviral Therapy	Type	Mechanism of Action	Mode of Administration	Efficacy Against MARV	Other Viral Targets	Regulatory Status
Favipiravir (T-705)	Nucleoside Analog	Inhibits RNA polymerase; induces lethal mutagenesis	Oral	Complete survival in mice when started 1–2 days post-infection. 83% survival in *cynomolgus macaques* with MARV infection.	Influenza strains (H1N1, H5N1, H7N9) Yellow fever Arenaviruses Flaviviruses Ebola virus	Licensed for influenza treatment in Japan; studied during 2014 EBOV outbreak
Galidesivir (BCX4430)	Nucleoside Analog	Targets RNA-dependent RNA polymerase (RdRp)	Intraperitoneally (IP); intravenously (IV)	100% survival in *cynomolgus macaque* when treated at 50 mg/kg.	Effective against other filoviruses like Ebola virus	N/A
Remdesivir (GS-5734)	Monophosphoramidate Prodrug	Inhibits viral RNA synthesis via vRNA-dependent RNA polymerase	Intravenously (IV)	83% survival rate with 10/5 mg/kg regimen in *cynomolgus* macaque monkeys; improved clinical outcomes and lower viral RNA levels in treated animals.	SUDV COVID-19 Hepatitis C Respiratory Syncytial Virus (RSV) Human Immunodeficiency Virus (HIV)	Authorized for treating COVID-19 in several countries
Obeldesivir (GS-5245)	Nucleoside Prodrug	Targets RNA-dependent RNA polymerase (RdRp)	Oral	Completely protected cynomolgus macaques when given once daily for 10 days after SUDV infection and protected 60% of animals when given for 5 days after infection.	SUDV COVID-19	N/A

**Table 2:** Treatment modalities for Marburg virus and their characteristics including the antibody type, time of administration, survival rate, and clinical outcomes.

Treatment Modality	Virus	Antibody Type	Timing of Administration	Survival Rate/Outcome
Polyclonal IgG treatment [43]	MARV	Polyclonal IgG	15–30 min after exposure, followed by additional doses at day 4 and day 8	100% protection (3/3 NHPs), with no clinical features and no viremia. MARV IgM response observed by day 4 and day 6 after challenge. IgG response increased and remained
Polyclonal IgG treatment [43]	MARV	Polyclonal IgG	48 h, followed by additional doses at day 4, day 8, and day 12	2/3 NHPs showed no clinical signs and completely cured; 1/3 NHPs showed mild clinical signs associated with high liver enzyme and low viremia, however fully recovered by day 16
Human monoclonal antibodies against MARV GP (MR-78-N; MR82-N; MR191-N) [44]	MARV, Ravn	Monoclonal antibody MR-78-N; MR82-N; MR191-N	2 days post-inoculation in guinea pigs	100% survival using MR-78-N and MR191-N, with virus not detected within plasma at 7 days post-infection; MR-82-N provided less protection and virus was detected within plasma
Monoclonal antibody MR191-N [44]	MARV	Monoclonal antibody MR191-N	4 days post-inoculation	100% survival, decline in viral load by day 7 post-inoculation with no evidence of virus by day 10
MR186-YTE [31]	MARV	Monoclonal antibody MR186-YTE	5 days post-inoculation single dose	100% survival (4/4 NHPs), all animals developed clinical features of disease; however, by day 16, no evidence of virus had been detected
Combination therapy of MR186-YTE and remdesivir [31]	MARV	MR186-YTE + remdesivir	6 days post-infection	80% efficacy

**Table 3:** Currently registered clinical trials for Marburg virus disease.

Trial ID.	Phase	Location(s)	Date	Estimated Completion Date	Purpose	Trial Name
NCT05817422	Phase 2	Uganda and Kenya	19 October 2023	1 May 2025	Vaccine	Monovalent chimpanzee adenoviral-vectored Marburg virus vaccine in healthy adults
NCT06620003	Phase 2	USA	January 2025	7/2026	Vaccine	A phase 2, randomized, double-blind, placebo-controlled trial to evaluate safety, tolerability, and immune responses of an investigational monovalent chimpanzee adenoviral-vectored Marburg virus vaccine in healthy adults
NCT03475056	Phase 1	USA	9 October 2018	19 December 2019	Vaccine	A phase 1, open-label study to examine the safety, tolerability, and immunogenicity of an investigational Marburg vaccine given by intramuscular (IM) injection to healthy adults. The study was a dose escalation of VRC-MARADC087-00-VP, a chimpanzee adenovirus serotype 3 (cAd3) vector vaccine, which encodes wild-type (WT) glycoprotein (GP) from Marburg virus
NCT06265012	Phase 1	USA	5 February 2024	16 September 2024	Vaccine	A phase 1 randomized, single-blind, placebo-controlled, ascending-dose study to evaluate the safety and immunogenicity of rVSV∆G-MARV-GP [Angola] (PHV01, Marburg virus glycoprotein [MARV GP] vaccine) in healthy adults. PHV01 is a live, attenuated rVSV vaccine expressing the MARV GP
NCT03800173	Phase 1	USA	10 December 2018	30 April 2019	Antiviral	A phase 1 double-blind, placebo-controlled, dose-ranging study to evaluate the safety, tolerability, and pharmacokinetics of galidesivir (BCX4430) administered as single doses via intravenous infusion in healthy subjects
NCT04723602	Phase 1	USA	6 January 2021	14 December 2021	Vaccine	A phase 1b trial to evaluate safety, tolerability, and immune responses of 2 monovalent chimpanzee adenoviral-vectored filovirus (Ebola-S and Marburg) vaccines to healthy adults, collection of plasma/serum for the purposes of assay development
NCT00997607	Phase 1	Uganda	February 2010	April 2012	Vaccine	A phase 1B study to evaluate the safety and immunogenicity of an Ebola DNA plasmid vaccine, VRC-EBODNA023-00-VP, and a Marburg DNA plasmid vaccine, VRC-MARDNA025-00-VP, in healthy adults in Kampala, Uganda
NCT00605514	Phase 1	USA	25 January 2008	21 June 2010	Vaccine	A phase 1 study to evaluate the safety and immunogenicity of an Ebola DNA plasmid vaccine, VRC-EBODNA023-00-VP, and a Marburg DNA plasmid vaccine, VRC-MARDNA025-00-VP, in healthy adults
NCT01353040		USA	May 2011	December 2011	Treatment	A randomized, double-blind, placebo-controlled, single-dose, dose-escalation study to assess the safety, tolerability, and pharmacokinetics of AVI-6003 in healthy adult volunteers
NCT02891980	Phase 1	USA	24 March 2017	21 March 2019	Vaccine	A phase 1 trial to utilize systems biology approaches to examine the safety, immunogenicity, and -omics response to MVA-BN(R)-Filo and Ad26.ZEBOV vaccines in healthy volunteers
NCT01566877		USA	May 2013	January 2014	Treatment	A randomized, double-blind, placebo-controlled, multiple-dose, dose-escalation study to assess the safety, tolerability, and pharmacokinetics of AVI-7288 in healthy adult volunteers

## Data Availability

All data produced during this study are included in the article.
